# A robust optimization model for multi-objective blood supply chain network considering scenario analysis under uncertainty: a multi-objective approach

**DOI:** 10.1038/s41598-024-57521-0

**Published:** 2024-04-24

**Authors:** Saeed Khakshouri Fariman, Kasra Danesh, Mostafa Pourtalebiyan, Zahra Fakhri, Ali Motallebi, Ali fozooni

**Affiliations:** 1 Department of Industrial Engineering, Eshragh Institute of Higher Education, Bojnourd, Iran; 2 Department of Electrical Engineering and Computer Science, Florida Atlantic University, Boca Raton, FL USA; 3 Department of Industrial Engineering, University of Science and Culture, Tehran, Iran; 4 Department of Management and Economics, University of Bergamo, Bergamo, Italy; 5 Faculty of Economics and Management, University of Tabriz, Tabriz, Iran; 6 Department of Marketing, University of Washington, Washington, USA

**Keywords:** Blood supply chain, HealthCare, Robustness, Disaster, Health care, Preclinical research, Stem-cell research

## Abstract

Annually, different regions of the world are affected by natural disasters such as floods and earthquakes, resulting in significant loss of lives and financial resources. These events necessitate rescue operations, including the provision and distribution of relief items like food and clothing. One of the most critical challenges in such crises is meeting the blood requirement, as an efficient and reliable blood supply chain is indispensable. The perishable nature of blood precludes the establishment of a reserve stock, making it essential to minimize shortages through effective approaches and designs. In this study, we develop a mathematical programming model to optimize supply chains in post-crisis scenarios using multiple objectives. Presented model allocates blood to various demand facilities based on their quantity and location, considering potential situations. We employ real data from a case study in Iran and a robust optimization approach to address the issue. The study identifies blood donation centers and medical facilities, as well as the number and locations of new facilities needed. We also conduct scenario analysis to enhance the realism of presented approach. Presented research demonstrates that with proper management, crises of this nature can be handled with minimal expense and deficiency.

## Introduction

Unexpected events, ranging from natural disasters to human-made crises, have a profound impact on human existence globally. These incidents have not only occurred recently in Iran but also in numerous other nations across the world. According to data, Iran ranked seventh among the top 10 countries for high-risk events globally. Over the past 20 years, natural disasters have killed, injured, or displaced up to 10% of Iran’s total population. One of the most damaging incidents was the Bam earthquake. A total of 21,000 blood units, or about 23 percent of the 106,000 total blood units given to earthquake victims, were lost in this disaster^1^. In contrast, the 2011 Great East Japan earthquake and tsunami resulted in an abundance of blood supplies^2^. The 2008 Iwate-Miyagi Nairiku earthquake raised concerns about the poor quality of the Chinese blood management system^3^. As demonstrated by these incidents, having an innovative and suitable blood supply chain program that can respond appropriately to a crisis in the future is unquestionably necessary. In such crises, control and effective management are essential.

**Figure 1:**
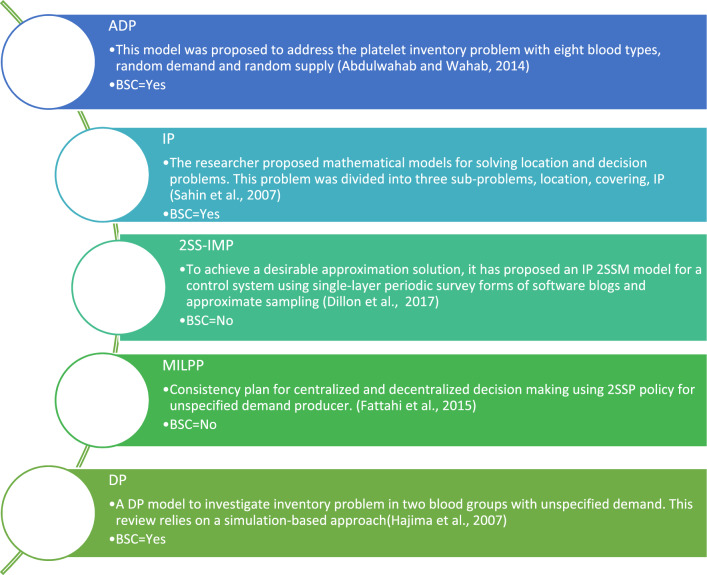
The review of framing methods.

Worldwide demand for blood donation and its byproducts, including platelets and plasma, has always been high ^53^. The significance of scale and relief efforts in blood transfusion management has been highlighted by global disasters, such as the 2008 China earthquake, the 2010 Haiti earthquake, the 2011 Japan earthquake and tsunami, and the 2013 Philippine Storm. Blood products are urgently needed by many wounded and injured persons; a lack of sufficient blood supplies in hospitals can exacerbate disasters^4^. According to the Energy Committee’s 2002 report, following the 9/11 attacks, more blood was collected than necessary. However, due to limited storage, a significant portion went unused. Managing and balancing the blood supply chain (SC) is crucial to meet demand and maintain benefits. The most popular blood derivatives are white blood cells (WBCs), packed red blood cells (RBCs), plasma (PLS), and platelets (PLT). All blood components deteriorate over time, RBCs can last up to 42 days, platelets several days, and plasma up to a year. As these blood components age, the risk of mortality from heart surgery increases, and studies show that RBCs older than 14 days may contribute to this risk^5^. The blood supply chain (BSC) involves collecting, testing, and distributing blood, which is essential for surgeries and emergencies. The issue of blood donation is global in scope. Stockpiling large amounts of blood leads to resource wastage and deterioration, while shortages result in human fatalities. The BSC must balance costs and casualties. Recent growth in healthcare studies shows increasing scholarly interest in this topic. Blood is a fundamental component of human existence. Critical steps in the blood transfusion process include collecting donated blood, transporting it to laboratories, manufacturing blood products, and delivering them to supply hubs like hospitals. These actions constitute the blood supply chains. Modeling optimization methods for the blood supply chain differs significantly from traditional modeling. Large blood donation facilities require sophisticated storage and maintenance equipment, which is costly. Blood testing is a crucial part of blood donation. Managing blood donation requires simultaneous strategic and tactical decision-making, such as locating and establishing mobile collection centers (MCCs) in specific areas, choosing laboratory facilities, securing various donors, designating various items, selecting the required centers, and shipping the blood bags. Costs in the supply chain include labor, wages, inspections, storage, spoilage, and distribution, with overall costs directly proportional to the provided survival probability^6^. Decision-making processes have been developed for every stage of the BSC due to challenges like demand unpredictability and the short shelf life of blood products. A mechanism must be in place to manage issues with the hospital blood supply chain and to respond appropriately to casualties. Given the humanitarian goals, many scientists and researchers aim to apply new knowledge-based enterprise designs to aid victims of natural or man-made disasters^7^. Xiang and Zhuang^54^ propose a novel victim-management model (Fig. 1).

The placement of the blood institution and blood transfusion are two operational and strategic considerations that must be made while designing the blood supply chain. Because the quantity of post-disaster blood demand might change over time, the blood chain’s architecture has to have a dynamic range^1^.

The paper makes several significant contributions to the field. Firstly, it incorporates the consideration of blood groups in the design of a blood supply chain network, an aspect that adds a layer of complexity and realism to the model. Secondly, it establishes the superiority of the proposed model over existing models in terms of both accuracy and effectiveness, marking a notable advancement in the field. Additionally, Kaveh and Ghobadi’s development of a Multistage Algorithm for Blood Banking Supply Chain in 2017 is highlighted, which focuses on evaluating the performance of the current distribution system across various blood centers. The results from this study underscore the efficacy of the proposed approach, particularly in terms of computational efficiency and overall performance.

Six sections are included in this paper to provide a comprehensive overview. An overview of the study’s background and significance is presented in the Introduction. The Literature Review provides an in-depth examination of existing research, highlighting key findings and gaps that this study seeks to fill. The Problem Statement identifies the specific issue or challenge the paper addresses. We present the findings of our research, supported by data and analysis, in the “Results” section. In the Discussion, these findings are interpreted and related to the literature and the problem statement. In the Conclusions, the study's main insights, implications, and potential directions for future research are summarized.

## Literature review

Sha and Huang^3^ suggested a multi-period approach with two stages of distribution and procurement to decide where to place facilities and how to distribute them to cut expenses. Additionally, after evaluating the research, it is clear that SSP2 is not commonly employed in the area of RBC inventory management. But we can research these techniques in papers like Gunpinar and Centeno’s^8^. For these policies, studies like Dillon et al.^9^ and Fattahi et al.^10^ employ random coding. The SSP2 two-step stochastic programming paradigm was created by Cunha. However, a theoretical stochastic programming model was created by Pauls-Worm et al.^11^. While ignoring the fact that these input parameters are uncertain and that all sources, needs, and input variables are ambiguous, none of the research address the multivariate property of blood labs. Figure 1 displays an analysis of framing techniques. This paper’s major goal is to offer a planning framework for improved event reaction so that we can minimize financial losses in the case of crises.

Rah et al.^55^ used the hierarchical analysis technique to thoroughly research factors influencing the placement of relief warehouses in logistics. The necessary commodities were identified by Su et al.^12^ after extensive research into emergency supplies at various stages of an event. Mete and Zabinsky^13^ offered a methodology for distributing and storing medical supplies in potential catastrophe management. They located the inventory and calculated the inventory level for each cluster using the model. This strategy has the benefit of using the underlying issue to direct vehicles to demand spots. Various earthquake scenarios are considered using this model in the Seattle-American Readiness and Response Phase. Ratkemper et al.^14^ proposed a methodology for distributing and allocating humanitarian supplies in response to requests. The two-objective concept aims to reduce both costs and unmet demand. In this model, the demand is split into two discrete and uncertain parts, and the model’s parameters are unknown. Rawls and Turnquist^15^ focused on distributing and identifying emergency supplies amid uncertainty. These procedures are divided into two stages: the first involves locating the commodities and determining how much inventory they can hold, and the second involves their transit across the network. In this model, the average transport distance should not be larger than a set value, and the probability of reaching a demand location should be at least a minimum threshold. This model has been applied to the South East Storm. Hsieh^16^ published articles on the blood supply chain under typical circumstances at two levels of supply and distribution. This study’s supply chain system includes hospitals, general blood centers, local blood centers, and donors. It sought to identify the number and location of general blood centers, their distribution to hospitals, and the level of availability in both general and local blood centers, aiming to reduce expenses and increase efficiency. Two models were created: one for the inventory control problem and the other for the allocation problem. The first model is a multi-objective one, and a Genetic Algorithm search was employed in Neighborhood Type II to solve the model. To reduce costs and delivery times, Fahimnia et al.^17^ introduced a two-element randomized model for the crisis supply chain. The Epsilon-Constrain technique and Lagrange method were used to find a hybrid solution to the issue. This approach can also be a useful tool for profit and cost analysis in the placement of facilities within the supply chain. Hosseinifard and Abbasi^18^ developed a network model to study the effects of inventory centralization on the long-term sustainability of the blood supply chain. This study examines the importance of inventory centralization in the second echelon of a two-echelon supply chain for perishable goods. The results indicate that centralization enhances the blood supply chain's sustainability. In blood supply chain inventory management, Dillon et al.^9^ proposed a novel approach to improve blood inventory replenishment control procedures. They employed two-stage stochastic programming to depict the ambiguous demand for blood. The results validate the technique and provide relevant management insights. Zahiri and Pishvaee^19^ developed a mathematical programming approach for blood supply chain network design.

Many scholars have found the idea of “green thinking” in supply chain management to be an intriguing subject. The document provides a periodical evaluation of the green supply chain^20^. By using Monte Carlo simulation, Sari^21^ offered a multi-criteria decision-making framework to assess green supply chain management strategies. Uncertainty in the supply chain network's architecture is still acknowledged as a major concern. A linear planning model was suggested by Peidro et al.^22^ to handle supply and demand uncertainty. Optimization methods are widely used in some studies such as supply chain management^23^, oil distribution^24^, drug delivery^25^. Little study has been done on the inventory and management of blood product supply chains, as demonstrated by Belien et al.^56^. Pierskalla^26^ provides four solutions by creating the ideal model: (1) where can I find blood banks? (2) How can I donate blood during a blood drive? (3) Locations providing blood donation services. (4) How is blood transferred?

To reduce uncertainty in the blood platelet supply chain, Fanoodi et al.^27^ studied the use of artificial neural networks (ANNs) and auto-regressive integrated moving averages (ARIMAs). Data from hospitals in Zahedan, Iran, were analyzed from 2013 to 2018. ANNs and ARIMA models outperformed the baseline model used by the Zahedan Blood Transfusion Center, especially when it came to predicting O+ and A+ platelet demand. To improve demand prediction in blood transfusion centers, these models should replace conventional statistical methods. Shirazi et al.^28,29^ reviewed recent advances in healthcare data mining, focusing on supervised and unsupervised learning paradigms. In this study, they categorized research papers based on their techniques and healthcare applications, emphasizing the importance of data mining in healthcare. A mathematical model for humanitarian supply chain planning in pandemic response was presented by Malmir and Zobel^30^. Taking into account transportation, delivery, and social costs, their model minimizes total costs. The use of this approach facilitates the organization of effective pandemic relief efforts. Shirazi et al.^28,29^ used big data and graph mining to detect disease areas using drug prescriptions. Based on expert opinion and the modularity metric, they identified six disease communities using the Louvain algorithm on a network of 50,000 Iranian prescriptions. Using game theory, Malmir et al.^31^ developed a model for supply chain quality management of hospital medical equipment. An analysis of firms' adoption of high-quality strategies in medical equipment supply was conducted by simulating the supply chain environment and analyzing the impact of reward and penalty systems.

We are searching for an appropriate model to do this while taking into account the expenses and limitations available and, on the other hand, taking into account the enhanced degree of reacting to the injured (see Table 1).

**Table 1:** A survey of related work.

Paper	Focus area	Methodology	Novelty	Comparison with our study
Presented paper	Blood supply chain optimization in post-crisis scenarios	Mathematical programming, robust optimization	Optimizes blood supply chains post-crisis, allocates based on facility needs, scenario analysis	Unique focus on post-crisis optimization and allocation based on facility location and demand
Khalilpourazari and Hashemi Doulabi^32^	Emergency blood supply chain network design	Flexible robust model, TLIR formulation	Addresses disruption and blood shelf-life, offers risk-averse solutions	Similar emphasis on robust solutions; different in handling disruptions and shelf-life considerations
Meneses et al.^33^	Blood supply chain planning optimization	Mathematical programming, OR-Modeling Framework	Reviews optimization in blood supply chain, offers a conceptual framework	Provides a broader conceptual framework; our study is more focused on practical implementation in crisis situations
Tirkolaee et al.^34^	Blood supply chain design during COVID-19	Bi-objective MILP model, socio-economic considerations	Incorporates pandemic impacts and socio-economic factors	Addresses pandemic context; presented study is tailored to natural disaster post-crisis scenarios
Momenitabar et al.^35^	Closed-loop blood supply chain design	Fuzzy multi-objective MINLP model, lateral resupply	Focuses on service level maximization, cost-saving through lateral resupply	Both aim for cost efficiency; different in approach with presented focused on post-crisis management
Eghbali et al.^36^	Blood supply chain design in disasters	Interval-valued fuzzy model, queue theory	Considers disruptions and queue theory for efficient design	Similar in disaster focus; different in employing fuzzy modeling and queue theory
Meneses et al.^37^	Decision-making models in crisis management	R-Modeling, MILP	Framework for developing decision-making models in crisis management	Different context: focuses more broadly on crisis management, while presented study is specific to blood supply chains
Lotfi et al.^38,39^	Waste recovery in supply chains	MWCND, HC, WPC	Analyzes impact of conservative coefficients on cost and risk	Different focus on waste recovery; presented study is specific to blood supply chain optimization
Kees et al.^40^	Optimization framework development	MILP, FMIGP	Framework for building decision-making models based on optimization	Similar in developing frameworks; presented study applies a specific model for post-crisis scenarios
Lotfi et al.^38,39^	Energy production and profit optimization	REL, DDRO, DDROMORELR	Relationship between ROR increase and energy production/profit	Different focus on energy and profit; presented study is centered on blood supply chain efficiency
Dorgham et al.^41^	Allocation of products to warehouses	THGs, Fuzzy chance-constrained programming	Focusing on customer demands and product allocation	Different context: focuses on general supply chain, while presented is specific to blood supply chains
Kazemi Matin et al.^42^	DMU performance and sustainability	BCCs, DDF	Impact of undesirable outputs on DMU performance and sustainability	Different focus on DMU performance; presented study is on optimizing blood supply in crisis scenarios
Lotfi et al.^38,39^	Cost, energy, and time optimization in supply chains	VCLSCND, EVaR	Algorithm's performance in cost, energy, and time optimization	Different focus on general supply chain optimization; presented study is specific to blood supply chains in crises

Various approaches and models have been introduced to tackle various challenges in blood supply chain management in recent years. Khalilpourazari and Hashemi^32^ developed a flexible, robust model for designing blood supply chains. They offer risk-averse, robust solutions based on their multi-objective Transportation-Location-Inventory-Routing (TLIR) formulation, which considers disruptions and blood shelf-life. A comprehensive review of the blood supply chain planning process was presented by Meneses et al.^33^. Using mathematical programming modeling, they presented a conceptual framework for planning blood supply chain management at the strategic and tactical levels. For the COVID-19 pandemic, Tirkolaee et al.^34^ presented a bi-objective Mixed-Integer Linear Programming (MILP) model. They also incorporated socio-economic factors and pandemic impacts into their Blood Supply Chain (BSC) model. In their paper, Momenitabar et al.^35^ proposed a fuzzy multi-objective Mixed-Integer Nonlinear Programming (MINLP) model with the goal of minimizing total network costs and maximizing service levels in hospitals. The case study demonstrated significant cost savings and improved service levels by incorporating lateral resupply strategies. A new interval-valued fuzzy mathematical model for designing blood supply chain networks considering disruptions was presented by Eghbali et al.^36^. A weighted multi-choice goal programming approach was used to manage uncertainties and optimize solutions.

## Problem statement

In this study, the supply and distribution issue is urgent and severe, so that expenses are kept to a minimum and, on the other hand, we won’t be as imperfect as feasible. We are dealing with a substantial rise in blood demand from the injured in the wake of the earthquake. The time scale is divided into four smaller time frames in order to analyze this, and demand is assessed in each frame independently. The first period lasts for 0–24 h, the second lasts for 24–72 h, the third lasts for the next 72 h, and the last lasts for the following week. In order to keep the expenses of the healthcare system and the ratio of shortages to demand to a minimum, it is important to decide how many temporary and permanent facilities should be developed or established, as well as where they should be located. Because they can’t simultaneously cut expenses and decrease flaws, these two objectives are incompatible. The challenge is complicated further by the large number of facilities involved in order to account for both the amount of supply and distribution. The topic being considered in this paper generally involves including numerous places, given that:

- Portable donuts and bots that can be positioned anywhere are examples of potential temporary blood sample facilities. These locations are in charge of gathering (providing) blood.
- Hospitals and clinics that are already in operation are examples of facilities that are now and potentially available for blood collection and treatment. A study area typically has a number of permanent facilities, and a prospective number can be taken into consideration for setting up. These locations are in charge of blood collection (supply) and treatment during the emergency (distribution).
- centers for blood donation, both current and future, that are located there. In a certain location, there are often several blood facilities, and adding more may be an option. Blood processing and testing take place at these sites.
- A location set aside for the care of injured persons houses potential field hospitals.
- The challenge is figuring out how to supply, process, and distribute blood while minimizing system costs and the ratio of shortages to demand. Temporary and permanent facilities must be developed or put in place, as well as their quantity and placement. Because they can't simultaneously cut expenses and decrease flaws, these two objectives are incompatible.

### Mathematical model

The best scenario is to have adequate short-term and long-term facilities spread out across several locations, as well as enough blood to prevent any blood shortages or treatment centers in the case of a crisis. However, since there are always financial restrictions, we must make plans to reduce expenses while still ensuring a sufficient and timely supply of blood. The issue thus comprises two objective functions, as was already indicated. Cost-cutting is the first goal function. There are costs associated with creating temporary facilities, permanent facilities (hospitals), field hospitals, erecting blood centers, and transporting blood from temporary and permanent facilities to the centers. Expenditures associated with blood, including blood costs from the Blood Center to the institution and blood testing and blood counts (Field Hospital and Permanent Facilitation). Definitively, with the goal of reducing waiting time and completion time costs.

**Figure:**
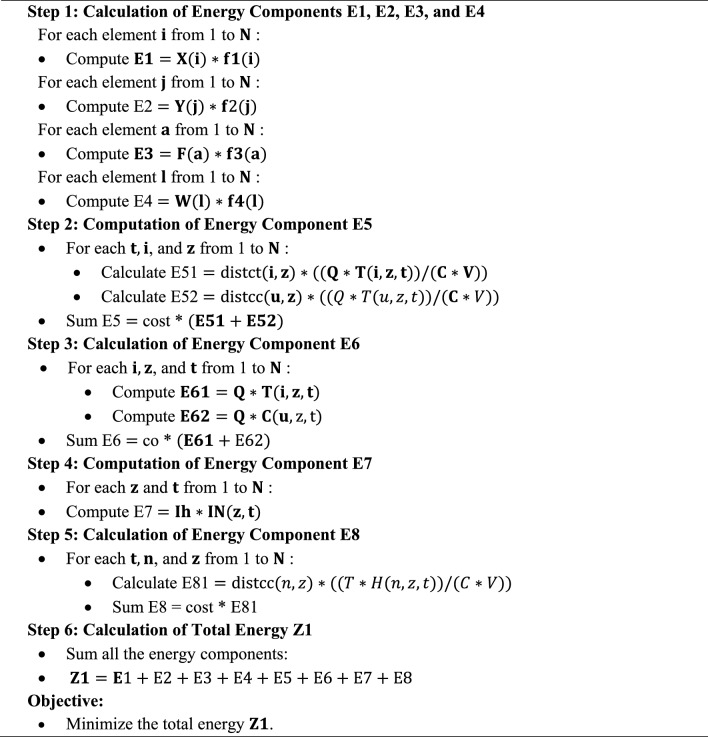
Algorithm 1: Objective 1

In Minimizing the deficit of blood flow during various times is the second aim function. In order to do this, we lower the ratio of shortages to demand throughout various time periods.

**Figure:**
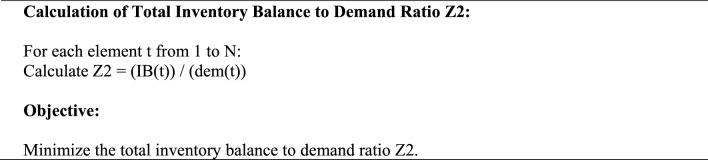
Algorithm 1: Objective 2

### Linearization

We encounter various limitations because of the limited capacity of blood centers for testing and processing, temporary and permanent facilities for blood collection, treatment, and maintenance, as well as the vehicle's capability for blood collection and distribution. We also encounter limitations when allocating blood donation facilities to blood centers in the supply sector and blood centers to treatment centers in the distribution region based on the locations of the facilities relative to one another. These are the restrictions.

1 $$ CH_{it} \le capa_{it} \forall i,t $$

2 $$ CH_{it} \le M \times X_{i} \forall i,t $$

3 $$ BH_{ut} \le capa_{ut} \forall u,t $$

4 $$ BH_{it} \le M \times Y_{j} \forall u \in j,t $$

5 $$ CH_{it} = \mathop \sum \limits_{z} QT_{izt} \forall i,t $$

6 $$ BH_{ut} = \mathop \sum \limits_{u} QC_{uzt} \forall u,t $$

7 $$ \mathop \sum \limits_{z} QT_{izt} + \mathop \sum \limits_{u} QC_{uzt} \le Capd_{zt} \forall z,t $$

8 $$ QT_{izt} \le M \times W_{l} \forall i,z \in l,t $$

9 $$ QC_{uzt} \le M \times W_{l} \forall u,z \in l,t $$

10 $$ QT_{izt} \le M \times X_{i} \forall i,z,t $$

11 $$ QC_{uzt} \le M \times Y_{j} \forall u,z,t $$

12 $$ \left( {\left( {\mathop \sum \limits_{i} QT_{izt} + \mathop \sum \limits_{u} QC_{uzt} } \right) \times \beta } \right) + IN_{zt - 1} - \mathop \sum \limits_{n} TH_{znt} + IN_{zt} $$

13 $$ \mathop \sum \limits_{z} TH_{znt} \le Capf_{n} $$

14 $$ \mathop \sum \limits_{z} \mathop \sum \limits_{n} TH_{znt} + IB_{t} = Dem_{t} \times \alpha $$

15 $$ TH_{znt} \le M \times W_{l} \forall i,z \in l,t $$

16 $$ TH_{znt} \le M \times Y_{j} \forall i,z \in l,t $$

17 $$ TH_{znt} \le M \times F_{a} \forall i,z,t $$

18 $$ \mathop \sum \limits_{z} \mathop \sum \limits_{n} TH_{znt} \ge \lambda \left( {Dem_{t} \times \alpha } \right) $$

Constraint (3); the i-th temporary facility's capability to capture blood should be less than or equal to the amount of blood collected there during each session. Constraint (4) Blood will be obtained in the event of brief relief in order to i-th assist the ionization. Constraint (5): For each period, the amount of blood collected in the u-th permanent facility must be less than or equal to its capacity for capture. Limitation (6) A permanent relief facility is in situ if the blood is drawn from a permanent facility. Constraint (7) states that the amount of blood delivered from the facility to the blood centers equals the amount of blood taken from the temporary vaccination i. Constraint (8) states that the amount of blood sent from the facility to the blood centers equals the amount of blood obtained from the permanent amenity. Constraint (9) states that the amount of blood that is transferred to the z-center of the blood must be less than or equal to its carrying capacity. If blood is transmitted from the temporary and permanent facilities to the blood center of l, the center of blood l is established, according to constraints (10) and (11), respectively. According to constraint (12), if blood is moved from temporary facility i-th to blood centers that have been made possible by temporary i. According to constraint (13) the permanent j-th facility has been constructed if blood is moved from the permanent facility of j to blood centers. According to constraint (14) the quantity of stored blood at the conclusion of each period is equal to the quantity of generated blood and the inventory of the preceding period less the quantity of blood supplied to the treatment facilities. Constraint (15) Any health center’s capacity should be matched by the volume of blood that is transmitted there. Constraint (16) Blood centers either meet blood requests or run out of blood. According to constraint (17), if the blood center l is built, blood from the center will be transported to treatment facilities. According to constraints (18) and (19), if the treatment center is treated for both j and a, blood from the z-center of blood is moved to the j-th therapeutic center. The quantity of demand demanded must be more than the equivalent of a percentage of the demand, according to constraint (20).

In some circumstances, the high mathematical model is taken into account. Since the crisis's intensity and circumstances were unpredictable, it was decided to simulate and examine the issue under a number of potential scenarios. Three scenarios with varied degrees of possibility were taken into account to investigate the issue. As a result, various circumstances result in variable setup costs for temporary facilities. Because of the crash that occurs following more severe crisis conditions, fees like excavation, shipping, and other expenses are also added to the launch price. In such a case, every facility makes an effort to make it easier for patients to be accepted in numbers greater than its full capacity. Due to this, the facility's capacity has altered depending on the situation. Additionally, if the facility is operating at full capacity, different circumstances will result in varying amounts of blood being collected from the blood donation center and transferred from the blood center to the treatment facility. Consequently, the scenario index is included in the model, and various scenario choice factors and variables change as a result, as indicated.

## Results

### Computational results

After presenting a scenario-based model for the issue and a suitable solution, we must gather data and statistical data. The Iranian province of Kermanshah is where this study was carried out. Information from the National Center for Earth Observations on the capacity of the temporary and permanent facilities, the center of blood from the current and ongoing facilities, as well as the distance between the facilities (Iranian Space Agency). The information gathered was used to address the issue with the Lingo software’s usage model as well as other issues. After resolving the issue, we were able to identify the instances we were looking for, as well as the temporary and permanent facilities that needed to be constructed and set up, as well as the best way to distribute blood units collected to the facilities and blood units produced in the treatment centers. The outcomes were subsequently examined in order to better analyze the model. In order to make the model more realistic, three situations have been taken into consideration. On November 21, 2017, at 21:48, a powerful earthquake with a Richter scale magnitude of 3.6 shook the provinces of Kermanshah, Ilam, and Kurdistan as well as large portions of the west and northwest of the nation. This earthquake was the most damaging to Sarpol-e-Zahab City because of its closeness to the city. More than 738 people died and the city was destroyed by the earthquake. With the exception of Iraq, this earthquake was also felt in Kuwait, Turkey, and Bahrain. There was a lot of devastation after the earthquake in Kermanshah, according to certain news sources; the amount of destruction was so great that the wreckage remained for months. However, the deputy governor general of Kermanshah claimed that there had been 100 fatalities in the Mehr housing complex, which he himself refuted on November 28, 2017. Its population in 2016 was 45, 481 according to the population and housing census (12,850 households).

**Figure 2:**
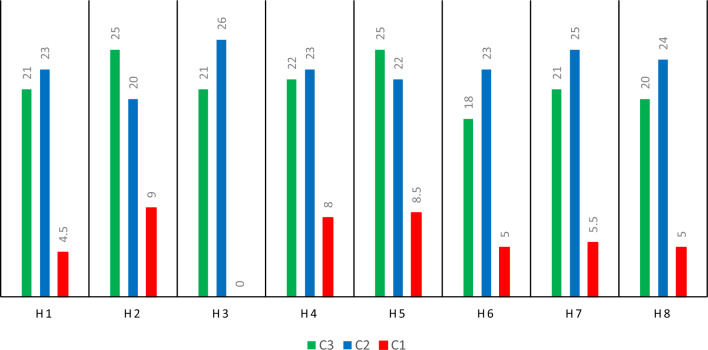
Distance between centers of blood from treatment centers (in km).

**Figure 3:**
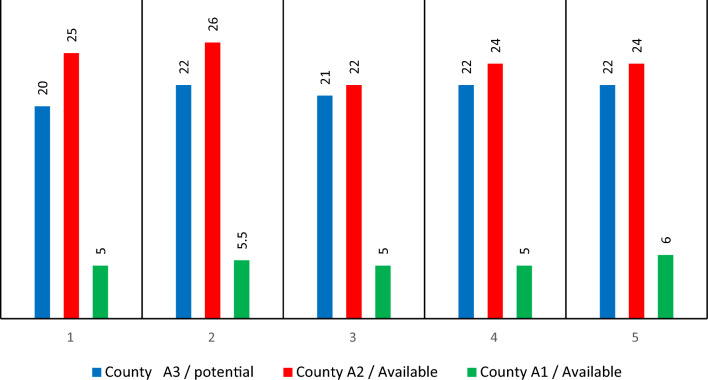
Distance of temporary facilities from blood centers (in km).

Three distinct intensity situations were taken into account in this investigation. Following situations are, correspondingly, harsher and demanding since the first scenario is less intense and naturally has less demand. Each scenario's likelihood of happening was estimated to be 0.15, 0.35, and 0.5%, respectively. Parentheses are used to separate each item. A blood facility is being built in Kermanshah, and the building cost is equal to one billion Rials. The separation between facilities is displayed in Figs. 2 and 3. These measurements are produced and gathered using Google Maps. Eight transient blood donations, five long-term blood donations and treatment centers, three blood centers, and five field hospitals were taken into consideration in this respect (Figs. 2 and 3).

Blood is donated for both ongoing and one-time blood drives. This institution sends the blood units it collects to blood banks. Blood units are examined and processed in the blood center to weed out unhealthy or diseased blood and get the blood ready for injection. Following testing and processing in the blood facility, it was determined that 0.8 percent of the blood was still accessible. This represents an average of statistics from several hospitals. Each unit of blood cost 450 ml, or 5000 Rials, for upkeep and testing. With specific vehicles, blood is transported to the treatment facilities. The distance unit was priced at 5000 Rials. Additionally, each truck can move 100 units of blood.

### Comparing scenarios

**Figure 4:**
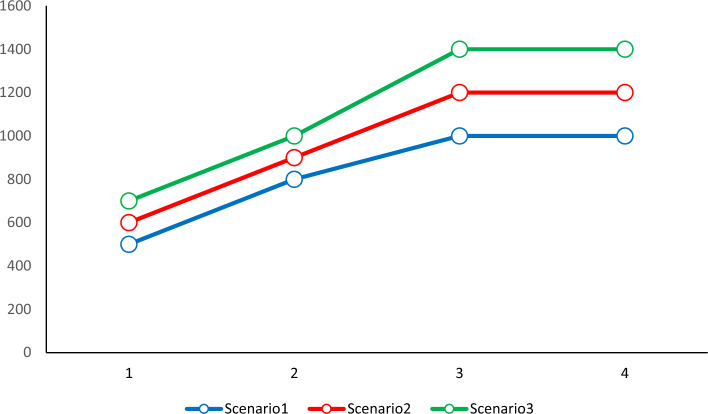
Demand for injured (people) different scenarios.

**Figure 5:**
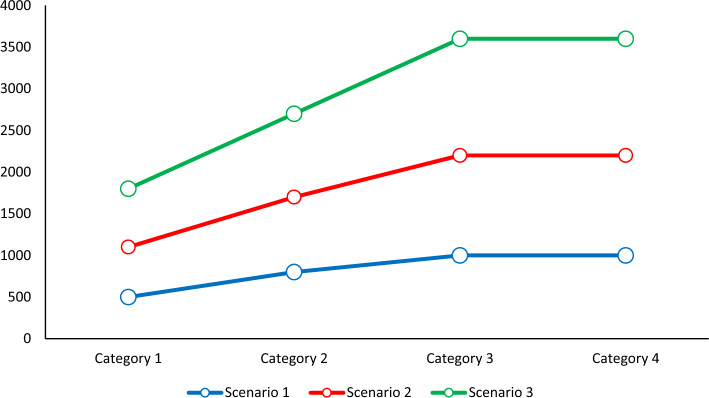
Demand for injured (people) different scenarios.

A significant portion of the injured in emergency scenarios require blood transfusions. The proportion of patients who require blood transfusions for therapy must be 0.8. The parameter, was set to 0.7 to determine the minimal quantity of demand. Figure 4 lists the need for wounded persons (Figs. 4 and 5).

**Figure 6:**
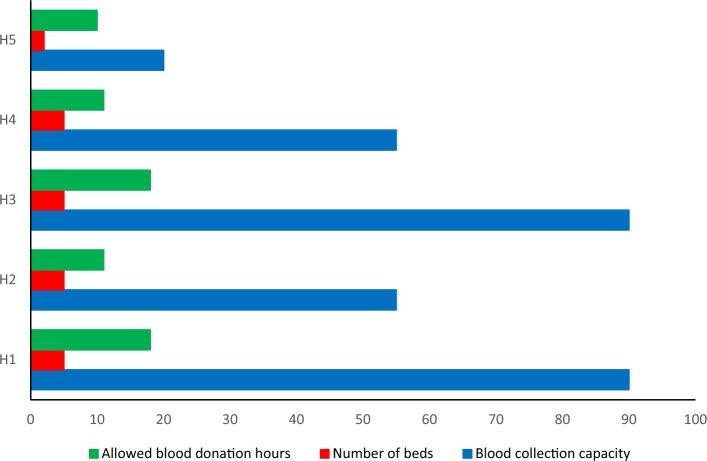
Calculation of blood capacity under the first scenario.

The facility’s capacity determines how much blood is stored there and how much is transferred to blood banks and subsequent treatment facilities. The information gathered was presented in Fig. 5 below in order to gather the relevant data on the capacity of facilities for permanent facilities in Kermanshah city. The blood donation capacity was determined by assuming that one person can donate blood on average once per hour, taking into account the treatment capacity of each hospital and the number of beds that are available in each one for blood donation. The capacity of each institution for blood transfusions per day is now determined by the number of beds available and the hours of donor referrals (Fig. 6).

**Figure 7:**
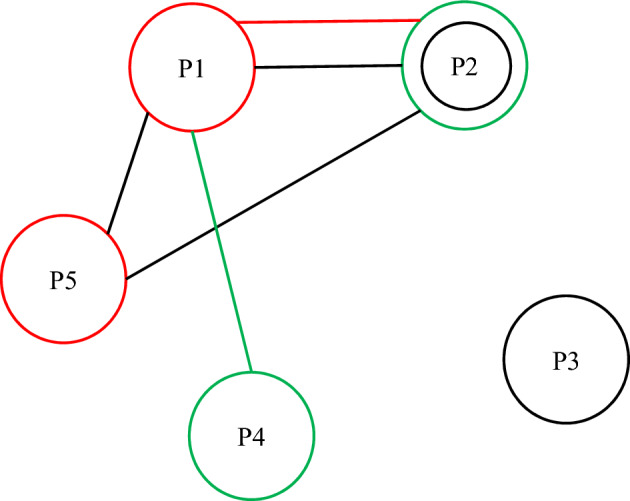
Number and location of temporary and permanent facilities to be set up and constructed, Black: Scenario 3, Green: Scenario 2, Red: Scenario 1, Circle is Blood centers, Lines are scenarios.

According to the findings, it is required to build up additional temporary facilities at certain places under various circumstances in addition to allowing temporary blood sample at the Place of Worker Street. Undoubtedly, the third scenario's more severe crisis has resulted in the development of additional temporary facilities. Additionally, at the chosen locations, there should be three permanent facilities: H3 Hospital, H4 Hospital, and H5 Clinic, in addition to the two permanent facilities for blood collection and treatment, H1 Hospital and H2 Hospital. Based on the findings, 6 to 8 new blood clinics in a rural city should be built in addition to those that already treat and distribute blood. Clearly, the worker’s street provides enough stable employment that there is no need to establish a field hospital. Due to the high expense of establishing facilities in this scenario, which was described in the preceding sections, there are fewer field hospitals in this scenario despite the great need in the third scenario and the absence of additional blood in this scenario. The scenario also aims to make the most of permanent facilities' capability. As a result, in this situation, the system has no inclination to build up more facilitations. Under the first, second, and third scenarios, the total quantity of blood shortage was 754, 28 and 887 units of blood, respectively. Considering the enormous volume of demand produced, this deficiency is relatively reasonable and sufficient (see Fig. 7).

**Figure 8:**
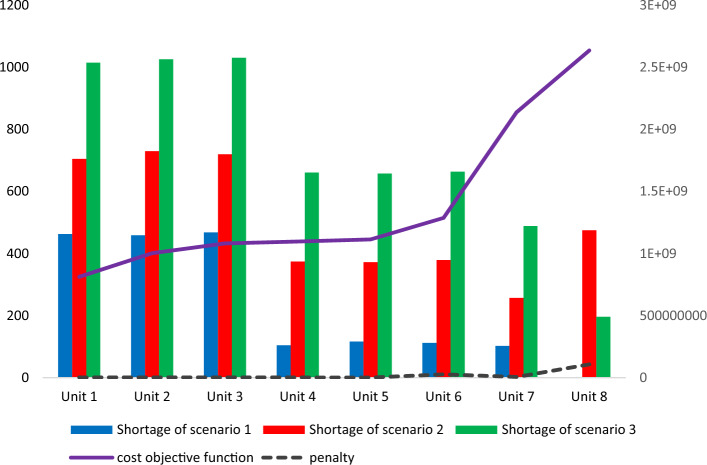
The amount of blood deficiency at the end of period t under the scenario z (blood unit).

The problem was resolved using several fines in a sophisticated optimization process to select the optimal fine amount. Figure 8 displays the cost and overall deficit for the four rounds and three IBB scenarios depending on various fines. The ideal fine level is one that strikes a reasonable balance between costs and overall deficits. The shortfall is lessened as the expenses rise by lowering the amount of fines. However, in the debate, limiting deficits takes precedence above minimizing expenditures. The greatest option for penalties is thus 1,450,000. Additionally, while the amount of the shortfall remains relatively constant, costs grow as the number of fines rises from 1,450,000 to higher. The chosen fine amount is therefore the best one that can be made.

The number of facilities, their locations, and the allocation of an ideal supply and distribution strategy for blood are determined using the data supplied in the preceding sections in the linear optimization model, and the issue is thus resolved. On a laptop with a 2.1 GHz Core i7 processor and 4 GB of RAM, the issue model was addressed using the Lingo 12 program. The problem contains two competing target functions, minimizing expenses and minimizing inadequacies, as was covered in the sections above. The software addressed each issue independently. Cost minimization, the first goal function, has a value of 1,031,243,000, while deficiency minimization, the second objective function, has a value of 16,071. The second objective function is given a greater priority than the cost objective function, making it the primary objective function as the model is now solved using the weighted approach, with the cost objective function acting as the constraint. Due to the usage of the weighted technique, in addition to the previously mentioned constraints, an additional constraint has been introduced, making this issue a single-objective problem. The cost objective function values are 24,923,620,124 Rials after the problem was solved using 26,710 the aforementioned manner, and the value of the shortage objective function climbed to 15,981. Table 2 displays the outcomes.

**Table 2:** Comparison of the results of problem solving separately and coherently.

	Shortage objective function	Cost objective function
Solve each issue individually	15,981	1,036,273,010
Problem Solving with weighted Method	26,710	24,923,620,124

## Discussion

Supply and demand are typically incompatible, donor blood supply is typically erratic and uncertain, and all of these factors contribute to the task's difficulty. Of course, a blood shortage might result in numerous fatalities and significant financial losses. The ambiguity of parameters in the actual world owing to their unpredictable nature is one of the most significant difficulties on the road. The care of this condition is a crucial concern since it has also produced blood that has only been utilized temporarily and has irreparable lesions (Belin & Forcé^56^). Today, one of the biggest problems facing the healthcare system is catastrophe management. The supply chain modeling technique has issues, including the very ambiguous decision-making environment^13^. Numerous investigations have been done to develop the proper relief supply roles in the blood supply chain^43^.

Some of the optimization algorithm that can be used for future works are as follows. Self-adaptive artificial fish swarm algorithm^44^, invasive weed optimization algorithm^45^, Robust bi‐level programming^46^, beam search algorithm^47^, Gradient-based grey wolf optimizer^48^. The basic components of BSC are illustrated by Osorio et al.^57^. Because of the unexpected increase in demand, it is also crucial to design a blood supply chain system^49^. For the emergency supply chain, several models have been put out in previous research. A brand-new theory on earthquake blood supply was put forth by Jabbarzadeh et al.^1^. Of fact, their primary goal was to reduce expenses. We shall explore a two-objective mathematical model whose component was time in Fahimnia et al.^17^. In the study of Zahiri and Pishvaee^19^, a model that reduces the supply chain's overall cost is demonstrated. Programming for several purposes was used to make this model. One of the most crucial elements in the sustainability of the blood supply was explored by Hosseinifard and Abbasi^18^ in relation to the effect of concentration on hospital stocks. According to their findings, fewer hospitals can help with transmission system flaws. For the design of the blood supply chain network, Cheraghi and Hosseini-Motlagh^50^ developed a mixed linear integer programming model. An automobile-selective routing model for blood collection was proposed by Sahinazan et al.^51^. Although there has been a lot of study on modeling, more has to be done on more useful models. For instance, no model can accurately depict how blood is transferred from the collection center to the major centers. These models for the blood supply chain can also take into account common decision-making processes, such as strategic, tactical, and operational planning^52^.

A problem is ultimately resolved utilizing the facts gathered and the best planned. The findings shown that we may meet this need for emergency blood while minimizing shortages and costs, and by building temporary and permanent facilities in selected places under various demand-related situations. Better management and control as well. Additionally, different scenario analysis settings had an impact on this issue, and the model was then updated with the results.

## Conclusions

Every year, numerous disasters affect regions across the globe, including earthquakes and floods. Given that individuals responsible for crisis management also oversee the lives of their citizens, managers in each country meticulously develop effective crisis management plans. This study examines the crisis supply chain in Kermanshah, one of Iran's earthquake-prone regions. Planning and preparedness are essential, as Iran ranks among the world's most disaster-prone nations. During emergencies, there is a critical need for blood products, in addition to supplying and distributing shelter, aid, and food supplies. The aim of this article is to devise a strategy for the prompt and efficient provision, processing, and distribution of blood. The challenges include determining the required number of temporary and permanent facilities and devising an optimal plan for blood preparation and distribution under varying conditions. This study employs a multifaceted linear programming model aimed at minimizing costs and disadvantages. The two objectives are in conflict; reducing one invariably diminishes the effectiveness of the other, with the deficit objective taking precedence due to its urgency and necessity. This consideration is integrated into the model-solving process. Additionally, Epsilon-constraint programming is used to find a multi-objective optimization solution. By considering three distinct scenarios, the study also attempts to align more closely with real-world situations. A case study was conducted in Kermanshah using actual data. The effectiveness of the approach is demonstrated through rapid problem-solving under various scenarios. Consequently, applying this method can be highly beneficial in emergency planning. Future work could explore the interactions between multiple echelons and facilities in uncertain environments, such as multi-echelons and multi-facilities, and utilize big data, blockchain, and other information technologies to predict and manage data in the blood supply chain.

## Data Availability

All data generated or analysed during this study are included in this published article.

## Index

*i*: Potential place of temporal intervention
*j*: Permanent facilitation (permanent hospital potential location)
*k*: Permanent facilitation (available hospital)
*a*: Placement of a potential field hospital
*b*: Available Blood Center
*l*: Potential Blood Center
*t*: The period
$u = j \vee k$: Permanent facilities set
$z = b \vee l$: Blood centers collection
$n = u \vee a$: Collection of treatment centers

## Parameters

$f1_{i}$: The cost of facilitating temporary imprisonment
$f2_{j}$: The cost of establishing permanent facilities j
$f3$: Hospital Launch Cost
$f4_{l}$: The cost of building a blood cell center
$Capc_{nt}$: The treatment capacity of the n-th treatment center
$capf_{n}$: The capacity to maintain blood at the n-th treatment center
$capb_{ut}$: Capacity for blood donation
$Capa_{it}$: Temporary ease of blood sampling
$capd_{zt}$: Capacity of the Z blood processing center
*Ih*: Maintenance costs for each unit of blood at the center of blood supply
*co*: Cost of testing each blood unit at the center of blood processing (blood processing)
*Cost*: Distance cost unit (per kilometer)
$Dem_{t}$: Demand for injured people for treatment during t
$\alpha$: The percentage of injured people needs blood products
$\beta$: Percentage of blood to be used after treatment at the Blood Center
$\lambda$: The percentage of blood product demand to be answered.
$disct_{iz}$: The distance between the interim facilitator i and the blood processing center z
$discc_{nz}$: The distance between the n center and the center of blood processing z
*CV*: Vehicle capacity for transporting blood

## Decision variables

$CH_{it}$: Blood collected in my temporary facilitator
$BH_{ut}$: Blood that is collected in the permanent ambulance.
$QT_{izt}$: Blood transmitted from the temporary relief i to the z-center of the t-th period.
$QC_{uzt}$: Blood transferred from the constant relief of u to the z center of blood during the t.
$TH_{znt}$: Bloods transmitted from the center of the blood z to the n center at the t-th time.
$IN_{zt}$: The amount of blood stored at the center of the blood z at the end of the t period.
$IB_{t}$: The amount of blood shortages in the t period
*Xi*: If temporary facility is created at the i-th location, otherwise
*Yj*: If a new permanent facility is created at the j-site, otherwise
*Wl*: If the new blood center is in place of l, otherwise
*Fa*: If the AH hospital is to be built, then this is the case
